# Evaluation of sperm mitochondrial function using rh123/PI dual fluorescent staining in asthenospermia and oligoasthenozoospermia^☆^

**DOI:** 10.1016/S1674-8301(10)60054-1

**Published:** 2010-09

**Authors:** Tiejun Zou, Xiang Liu, Shangshu Ding, Junping Xing

**Affiliations:** Department of Urology, the First Affiliated Hospital of School of Medicine of Xian Jiaotong University, Xian 710061, Shaanxi Province, China

**Keywords:** asthenospermia, oligoasthenozoospermia, mitochondrial membrane potential, flow cytometry, Rhodamine 123/propidium double fluorescent staining

## Abstract

**Objective:**

The recent advent of flow cytometry (FCM), coupled with fluorescent dyes, has been successfully applied to assess mitochondrial function. The aim of this study was to investigate the feasibility and clinical significance of detecting sperm mitochondrial function and to evaluate sperm mitochondrial function by using Rhodamine 123/propidium (Rh123/PI) dual fluorescent staining and FCM in asthenospermia and oligoasthenozoospermia.

**Methods:**

Twenty-five fertile men (with normal sperm parameters) and 230 infertile patients were examined. Fifty-five patients of the above 230 patients were selected for idiopathic infertility samples and were divided into two groups: asthenospermia (*n* = 30) and oligoasthenozoospermia (*n* = 25). Rh123/PI dual fluorescent staining and FCM were carried out to examine sperm mitochondrial function.

**Results:**

Significant differences were found between the normal and abnormal semen samples (*P* < 0.05) when Rh123^+^/PI^−^, Rh123^−^/PI^+^ and Rh123^−^/PI^−^ sperm were examined by FCM, but there was no significant difference between the asthenospermia (*P* = 0.469) and oligoasthenozoospermia group (*P* = 0.950) when Rh123^+^/PI^−^ and Rh123^−^/PI^+^ sperm were then examined; however, a significant difference was found between the 2 groups (*P* = 0.003) when Rh123^−^/PI^−^ sperm were examined. There was no correlation between Rh123^−^/PI^−^ sperm and semen parameters in the normal group, but there was a significant negative correlation between the sperm concentration and Rh123^−^/PI^−^ sperm in asthenospermia and oligoasthenozoospermia patients (*r* = -0.509, -0.660; *P* = 0.018, 0.038).

**Conclusion:**

Rh123/PI dual fluorescent staining and FCM can provide reliable information to assess the quality of sperm and reveal differences in mitochondrial membrane potential in asthenospermia and oligoasthenozoospermia.

## INTRODUCTION

The structural and functional integrity of the plasma membrane and mitochondria, as well as motility are critical to fertilization by sperm[1]. The percentage of motile sperms is the most common factor used to evaluate sperm quality. Generally speaking, microscopic and physicochemical examinations of semen specimens are routinely used for the diagnosis of male infertility. These analyses of semen included the measurement of spermatozoon concentration, morphology and motility. Although this conventional analysis of semen provides considerable information, it is limited in predicting male fertility. In recent years, flow cytometry (FCM) has been employed extensively to study various attributes of sperm, because this technique can automatically analyze defined parameters of several thousand cells at the single-cell level within a few min[2], as well as provide statistically reliable data[3]-[8].

Mitochondria in dead cells are readily distinguishable from those in live cells as they are not able to retain Rh123 (Rhodamine 123) after washing. Cell membrane integrity can be evaluated by staining sperm with propidium iodide (PI), a fluorescent dye that binds with DNA. The intact membrane of viable sperm will prevent PI from entering the sperm to bind with cellular DNA. In contrast, damaged membrane of dead/moribund sperm allows PI to enter and bind with DNA to produce red fluorescence. Dual fluorescent staining (Rh123/PI) combined with FCM has been developed for simultaneous determination of mitochondrial membrane potential (MMP) and membrane integrity of sperm[2],[9]-[11]. In other assays mainly based upon the use of DNA intercalating fluorescent compounds, acridine orange and ethidium bromide have been used to evaluate the presence and the number of such cells by FCM[12]-[13].

The movement of spermatozoa depends on the energy supplied by mitochondria at the middle piece of the tail. Thus, the examination of mitochondrial function may be helpful in the diagnosis and evaluation of male infertility. Previously, sperm examination usually only concentrated on the changes of sperm morphology and movements. Our aim of this study was to investigate the feasibility and clinical significance of assessing sperm mitochondrial function in asthenospermia and oligoasthenozoospermia using Rh123/PI dual fluorescent staining and FCM.

## MATERIALS AND METHODS

### Collection and classification of clinical materials

All the semen specimens were collected at the First Affiliated Hospital of Xian Jiaotong University School of Medicine from Aug, 2009 to Mar, 2010. Twenty-five fertile men (with normal sperm parameters) and 230 infertile patients were examined. One hundred and seventy five subjects were excluded because of case questionnaire and/or physical examination. Fifty-five patients of the above 230 patients were selected for idiopathic infertility samples based on semen examination and infertility factors, such as immune factors (anti-spermatozoal antibody IgG and IgA), infection (mycoplasma, chlamydia) and biochemical abnormalities (seminal α-1, 4-glucosidase, acid phosphatase and fructose assays). Relating to the above infertility factors, mycoplasma was detected by improved cultivation of semen samples; immune factors were examined in sera by enzyme-linked immunosorbent assay (ELISA) kits from Xindi Company (Nanjing, China), and α-1, 4-glucosidase, acid phosphatase, and fructose were determined by routine biochemical assays. The protocol was approved by the Clinical Research Ethical Committee of Xian Jiaotong University, China. Idiopathic infertility patients were divided into two groups: the asthenospermia group (*n* = 30) and the oligoasthenozoospermia group (*n* = 25). According to the WHO criterion[15], all 80 semen samples including 25 samples in the control group were collected into clean container by masturbation after 3-7 d of sexual abstinence. The asthenospermia group: The age of the 30 patients ranged from 25-39 (31.6±4.8) y, less than 50% of the spermatozoa moved straight [(a+b)%] or less than 25% of the spermatozoa moved rapidly straight, sperm concentration was more than 20×10^6^/mL, and other parameters were similar to those observed in normal semen. The oligoasthenozoospermia group: The age of the 25 patients ranged from 23-37 (28.3±4.1) y, and asthenospermia was combined with a sperm concentration of less than 20×10^6^ spermatozoa/mL, or less than 40×10^6^ spermatozoa were ejaculated at one time[14]. The control group: The age of the 25 healthy males ranged from 23-36 (28.2±3.7) y. Their physical examinations were normal and the semen met the WHO criterion[15]. There was no difference in age of the 80 patients by *q*-test and variance analysis (*P* > 0.05).

### Conventional semen analysis

Ejaculates were allowed to liquefy at 37°C for 30 min. When liquefaction occurred, each specimen was divided equally into 2 parts: One part was assessed by light microscopy and computer assisted sperm analysis (CASA, WLJY2 9000, Weili Company, China) according to the procedure proposed by the WHO[16], which included the measurement of spermatozoon concentration, morphology, and motility. Four types of motility were assessed and classified as follows: a) rapid progressive or linear motility; b) slow progressive or curvilinear motility; c) not progressive or *in loco* motility; d) lack of motility. The second part of the sample was used for Rh123/PI double fluorescent staining followed by FCM.

### Rh123/PI double fluorescent staining and FCM examination

Semen samples were first washed with phosphate-buffered saline (PBS: 0.01 mol/L, pH 7.4) and then centrifuged (4000 *g*, 30 s) twice. PBS was added to wash the sperm and the concentration of spermatozoa was adjusted to 5×10^6^/mL. Rh123 (Sigma, USA) was then added to a final concentration of 5 µg/mL, and the sample incubated for 5 min at 37°C in the dark. The spermatozoa were next washed with dye-free PBS to eliminate non-specific binding of dye to the mitochondria, and centrifuged again. PI (Sigma) was added to a final concentration of 5 µg/mL and the sample was incubated for 5 min at 37°C in the dark. Finally, the sample was resuspended in PBS and immediately examined by FCM (FACSCalibur, BD, USA)[17]. The excitation wavelength was set to 480 nm, and application of side scatter (SSC) and forward scatter (FSC) was linearly amplified, and logarithmic amplification was performed for fluorescence channel FL1 (green) and FL2 (red). The fluorescence densities of Rh123 and PI of 10,000 spermatozoa in each semen sample were examined and recorded. The results obtained by Rh123 staining was expressed in terms of changes in fluorescence intensity, so that MMP variations were identified as shifts of the mean fluorescence peak in FL1. For the simultaneous evaluation of cell viability and MMP, sperm cells were counterstained with PI, the emission of which was analyzed in FL2.

### Statistical analysis

All the data were expressed as mean±SD. The Kruskal-Wallis one-way analysis-of-variance-by ranks test and one-way ANOVA were used to determine the results of conventional semen parameters and FCM, and their correlations were analyzed with the Spearman correlation test and Pearson rank correlation analysis, where appropriate. All statistical analysis was performed with SPSS13.0 software (SPSS Inc., USA), and *P* < 0.05 was considered statistically significant.

## RESULTS

### Sperm double fluorescent staining by Rh123/PI

**Fig. 1:**
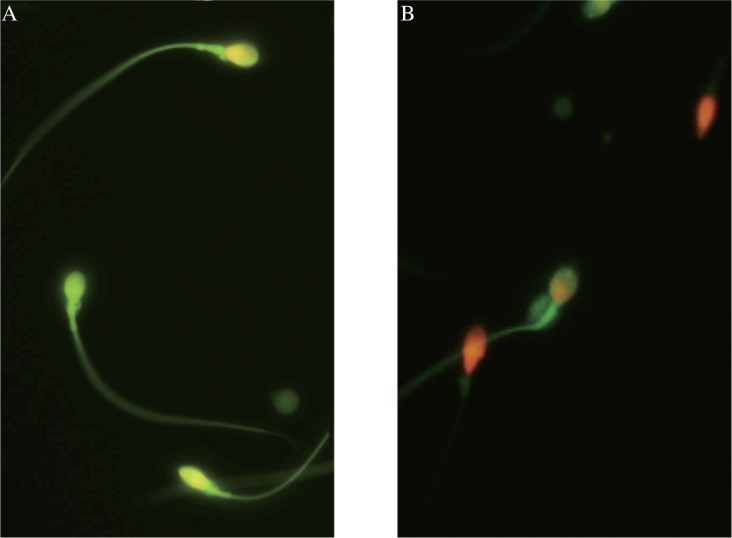
Sperm fluorescent staining. A: After staining with Rh123, green fluorescence was centralized on the sperm body under fluorescence microscope, suggesting that the mitochondria functioned well (×400); B: After double fluorescent staining with Rh123/PI, dead sperms from asthenospermia patients were marked by red heads (×400).

As shown in ***Fig. 1***, under the fluorescence microscope (BX51, Olympus, Japan), sperms with normal mitochondrial function stained by RH123 exhibited green tail (***Fig. 1A***). Sperm stained by double fluorescent dyes Rh123/PI were marked by a green body or a green body and a red head (***Fig. 1B***). Sperm exhibiting a green body and a red head indicated that, although mitochondrial function was normal, there was an increase in cell membrane permeability. Spermatozoa exhibiting only a red head indicated dead sperms.

**Fig. 2:**
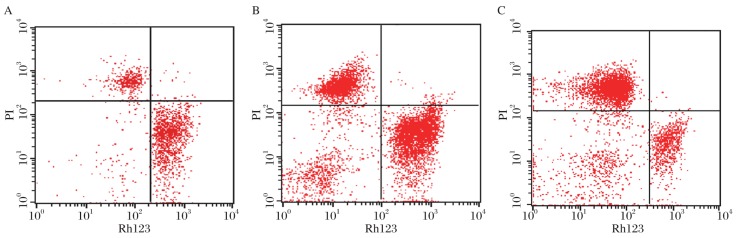
Results of sperm FCM. The lower right quadrant of each graph, Rh123^+^/PI^−^, shows the sperm with normal mitochondrial function; the lower left quadrant shows Rh123^−^/PI^−^, which means the sperm losing mitochondrial function; the upper left quad-rant, Rh123^−^/PI^+^, shows necrotic sperm. A: normal seminal sample. B: the asthenospermia group. C: the oligoasthenozoospermia group.

By FCM, sperms stained by Rh123/PI were easily divided into four parts (***Fig. 2***): the lower right quadrant of each graph, Rh123^+^/PI^−^, shows sperms with normal mitochondrial function; the lower left quadrant contains Rh123^−^/PI^−^ sperms that have lost their mitochondrial function; the upper left quadrant, Rh123^−^/PI^+^, shows necrotic sperms. Thus, sperm double fluorescent staining by Rh123/PI can readily distinguish mitochondria in dead and live cells. It is by FCM that several thousand sperms can be automatically analyzed at the single-cell level within a few minutes.

As shown ***Fig. 2***, sperms in normal group were centered in the lower right quadrant of each graph. It means that sperms with good mitochondrial function make up the greater part of all sperms in normal group. However, sperms chiefly focus on the left quadrant of each graph in the asthenospermia group and oligoasthenozoospermia group. It indicates that the proportion of sperms losing mitochondrial function and necrotic sperms was increased in both groups, especially in the oligoasthenozoospermia group.

### Sperm staining differences among the three groups

As shown in ***Table 1***, there were significant differences in the percentage of Rh123^+^/PI^−^, Rh123^−^/PI^−^ and Rh123^−^/PI^+^ sperm between any two of the three groups (all *P* < 0.05). There was a significant difference in the percentage of Rh123^−^/PI^−^ sperm (*P* = 0.003), but not the Rh123^+^/PI^−^ or Rh123^−^/PI^+^ sperm (*P* = 0.469, 0.950, respectively) between the asthenospermia and oligoasthenozoospermia group. As shown in ***Table 2***, no correlation was found between FCM results and CASA parameter data of Rh123^−^/PI^−^ sperm in the control group, while a negative correlation was found between FCM results of Rh123^−^/PI^−^ sperm and sperm concentration in the asthenospermia and oligoasthenozoospermia group (*r* = -0.509, -0.660; *P* = 0.018, 0.038).

**Table 1:** Comparison of FCM results among control, asthenospermia and oligoasthenozoospermia patients

Group	Rhl23^+^/PI^−^	Rhl23^−^/PI^−^	Rhl23^−^/PI^+^
Normal semen subjects (*n* = 25)	57.34 ± 10.54	10.76 ± 5.83	29.71 ± 7.77*
Idiopathic asthenospermia semen subjects (*n* = 30)	38.29 ± 16.83^a^	15.15 ± 10.12^a^	44.67 ± 17.81^a^
Idiopathic oligoasthenozoospermia semen subjects (*n*= 25)	33.51 ± 21.45^b^	33.36 ± 24.46^b,^ ^c^	29.27 ± 13.52 ^b^

*The data was NOT considered to obey normal distribution and analyzed with the Kruskal-Wallis one-way analysis-of-variance-by-ranks test. Among the control, asthenospermia and oligoasthenozoospermia group, significant difference can be found between any 2 of the 3 groups (*P* < 0.05). a: asthenospermia *vs* control group (*P* < 0.05). b: oligoasthenozoospermia *vs* control group (*P* < 0.05). c: asthenospermia *vs* oligoasthenozoospermia group (*P* < 0.05).

(mean±SD, %)

**Table 2:** Comparison of correlation between FCM results of 80 specimens and their parameters.

Parameters	*N*	Rhl23^+^/PI^−^		Rhl23^−^/PI^−^		Rhl23^−^/PI^+^	
		*r*	*P*	*r*	*P*	*r*	*p*
a(%)	80	0.514*	0.001*	-0.470	0.002	-0.070	0.667
b(%)	80	0.569*	0.000*	0.200	0.215	-0.294	0.065
c(%)	80	0.426*	0.006*	-0.180	0.266	-0.166	0.305
d(%)	80	-0.587	0.000	0.361	0.022	0.274	0.087
a+b(%)	80	0.671*	0.000*	-0.385	0.014	-0.251	0.118
Motility	80	0.587	0.000	-0.361	0.022	-0.274	0.087
Volume	80	-0.015	0.925	0.042	0.796	-0.096	0.556
Concentration	80	-0.352	0.026	-0.673	0.000	0.191	0.237
Time	80	-0.123	0.450	0.043	0.794	0.001	0.995

*The data match normal distribution, the Pearson rank correlation analysis was performed, and Spearman correlation test was performed for the others. a: rapid progressive or linear motility. b:slow progressive or curvilinear motility. c: not progressive or in loco motility. d: lack of motility.

Statistical differences in the distribution of Rh123^+^/PI^−^, Rh123^−^/PI^−^ and Rh123^−^/PI^+^ sperm were found between the asthenospermia and control group, and also between the oligoasthenozoospermia and control group (*P* < 0.05). The percentage of Rh123^+^/PI^−^ sperm (normal sperm) in the control group (57.34% ±10.54%) was much higher than that in the asthenospermia (38.29%±16.83%) and oligoasthenozoospermia group (33.51%±21.45%), while the percentage of Rh123^−^/PI^−^ sperm (10.76%±5.83%) and Rh123^−^/PI^+^ sperm (29.71%±7.77%) in the control group were much lower than that in the asthenospermia (15.15%±10.12%, 44.67%±17.81%) and in the oligoasthenozoospermia group (33.36%±24.46%, 29.27%±13.52%). There was no significant difference in the percentage of Rh123^+^/PI^−^ or Rh123^−^/PI^+^ sperm between the asthenospermia and oligoasthenozoospermia group (*P* = 0.469, 0.950), indicating that the number of normal and dead sperms was similar in the asthenospermia and oligoasthenozoospermia group. There was a significant difference in the number of Rh123^−^/PI^−^ sperm between the asthenospermia and oligoasthenozoospermia group (*P* = 0.003, ***Table 1***), showing that more sperm in the oligoasthenozoospermia group exhibited dynamic-deficient movement than the asthenospermia group.

No correlation was found between Rh123^−^/PI^+^ sperm and their CASA parameter (*P* > 0.05), but correlations were observed between Rh123^+^/PI^−^ sperm and their CASA parameters except for the semen volume (*P* = 0.925) and liquefaction time (*P* = 0.450). For example, Rh123^+^/PI^−^ was negatively correlated with sperm concentration (*r* = -0.352, *P* = 0.026) and lack of motility (%) (*r* = -0.587, *P* = 0.000), but positively correlated with other parameters, and Rh123^−^/PI^−^ sperm were negatively correlated with sperm concentration (*r* = -0.673, *P* = 0.000), activity (a %) (*r* = -0.470, *P* = 0.002), but positively correlated with activity, d (%) (*r* = 0.361, *P* = 0.02, ***Table 3***).

**Table 3:** Comparison of correlation between FCM results of 30 asthenospermia specimens and their parameters.

Parameters	*n*	Rhl23^+^/PI^−^		Rhl23^−^/PI^−^		Rhl23^−^/PI^+^	
		*r*	*P*	*r*	*P*	*r*	*P*
a(%)	30	0.598*	0.001*	-0.290	0.203	-0.487*	0.025*
b(%)	30	0.472*	0.031*	0.070	0.763	-0.631*	0.002*
c(%)	30	0.501*	0.021*	0.039	0.867	-0.581*	0.006*
d(%)	30	-0.607	0.004*	0.013	0.955	0.668*	0.001*
a+b(%)	30	0.617*	0.003*	-0.064	0.784	-0.664*	0.001*
Motility	30	0.607	0.004*	-0.013	0.955	-0.668*	0.001*
Volume	30	0.084	0.719*	0.167	0.469	-0.119*	0.607*
Concentration	30	0.332	0.141	-0.509	0.018	-0.130	0.575
Time	30	-0.289	0.204	0.117	0.613	0.015	0.948

^*^ The data match normal distribution, the Pearson rank correlation analysis was performed, and Spearman correlation test was performed for the others. a: rapid progressive or linear motility. b: slow progressive or curvilinear motility. c: not progressive or in loco motility. d: lack of motility.

## DISCUSSION

Male infertility is a common clinical disease and the cause for many cases is unknown. According to a study by Curi *et al.*[18], about 19% of male infertility is due to asthenospermia. In the past, it was recognized that there were limitations in the conventional methods for predicting male fertility. Therefore, many newer techniques have been developed to increase the accuracy, speed and discriminating power of semen analysis. FCM seems to be a reliable tool for improving the diagnosis of male infertility[8]. Reduced sperm activity is characteristic of asthenospermia and oligoasthenozoospermia. The energy supply of sperm mainly depends on the tri-carboxylic acid (TCA or citric acid or Krebs) cycle in the mitochondria. When the energy generated by the TCA cycle is conducted along the respiratory chain, protons are pumped out from the matrix inside the mitochondrial inner membrane, forming MMP. Thus, the MMP reflects the TCA cycle activity and the function of mitochondria. Mitochondria provide energy for sperm to swim toward the oocyte and penetrate its zona pellucida[19]. Hence, any damage to mitochondrial function could impair sperm motility and fertilization.

As fluorescent dyes, Rh123 and PI can be used to evaluate the function of mitochondria and nucleus, respectively[20]. Rh123 crosses the mitochondrial membrane and resides in the mitochondria, and green fluorescence is detected from the inside of mitochondria. When MMP disappears under pathologic conditions, Rh123 does not move into the mitochondria. In dead cells, PI crosses the plasma membrane and combines with nuclear DNA, producing red fluorescence. Rh123/PI double fluorescent staining can distinguish dead sperm (Rh123^−^/PI^+^ sperm), live sperm with an impaired MMP (Rh123^−^/PI^−^ sperm) and normal sperm (Rh123^+^/PI^−^ sperm)[21].

Using the WHO guidelines, Wu *et al.*[22] classified sixty-three semen samples only as normal and abnormal groups and drew similar conclusions. In our study, the FCM results showed that the functional levels of mitochondria in the asthenospermia and oligoasthenozoospermia groups were different. The d (%) value was negatively correlated with Rh123^+^/PI^−^ sperm, but positively correlated with Rh123^−^/PI^+^ sperm, while a (%), b (%), c (%) and a+b (%) values were totally different from d (%) values. The d (%) can be divided into dead Rh123^−^/PI^+^ sperm and MMP deficient Rh123^−^/PI^−^ sperm when examined by FCM. The statistical difference in Rh123^−^/PI^−^ sperm in the asthenospermia and oligoasthenozoospermia suggests that the energy supply may be the main difference between the two groups.

It is difficult to clarify the causes of damage to the mitochondria in asthenospermia and oligoasthenozoospermia. Varicocele has been implicated as a major cause of male infertility[23]. Studies have shown that varicocele caused an increase in sperm intracellular superoxide levels and a decreased MMP and other parameters in both testicles in a rat model. This study proposed that one of the main sources of ROS production in varicocele is intracellular, which in turn suggested that the elevation of intracellular ROS could be one of the important mechanisms responsible for mitochondrial and tissue damage[24],[25]. In addition, environmental factors may also be very important. The membrane integrity of sea urchin sperm was impaired by environmentally relevant levels of UVB but not UVA, suggesting that UV-induced ROS could damage sperm motility and fertility by attacking the mitochondria (for UVA and UVB) and plasma membrane (for UVB, but not UVA)[26].

Presently, sperm quality is evaluated by conventional semen analysis, determining concentration, motility and morphology using light microscopy. However, the introduction of clinical assisted reproduction a quarter of a century ago required the isolation of motile spermatozoa. As the indication of assisted reproduction shifted from mere gynaecological indications to andrological indications over the years, this prompted andrological researchers to understand the physiology of male germ cells better and develop more sophisticated techniques to separate functional spermatozoa from those that are immotile, have poor morphology or are not capable of fertilizing oocytes. Rh123/PI dual fluorescent staining and FCM revealed a difference in MMP in asthenospermia and oligoasthenozoospermia, and the MMP data represented an important method of determining the quality of sperm in asthenospermia and oasthenozoospermia and sperm preparations of infertile men, aiding in predicting successful *in vitro* fertilization[27],[28].

In conclusion, Rh123/PI dual fluorescent staining and FCM analysis can provide reliable information to assess the quality of sperm and reveal difference in MMP in asthenospermia and oligoasthenozoospermia.

## References

[1] He SY, L Curry Woods III (2004). Effects of dimethyl sulfoxide and glycine on cryopreservation induced damage of plasma membranes and mitochondria to striped bass (Morone saxatilis) sperm. J Cryobiol.

[2] Graham JK, Kunze E, Hammerstedt RH (1990). Analysis of sperm cell viability, acrosomal integrity, and mitochondrial function using flow cytometry. J Biol Reprod.

[3] Tao J, Du J, Critser ES, Critser JK (1993). Andrology: Assessment of the acrosomal status and viability of human spermatozoa simultaneously using flow cytometry. J Hum Reprod.

[4] D'Cruz OJ, Haas GG (1996). Fluorescence-labeled fucolectins are superior markers for flow cytometric quantitation of the human sperm acrosome reaction. J Fertil Steril.

[5] Glander H J, Schaller J (1993). Beta 1-integrins of spermatozoa: A flow cytophotometric analysis. Int J Androl.

[6] Nikolaeva MA, Kulakov VI, Ter-Avanesov GV, Terekhina LN, Pshenichnikova TJ, Sukhikh GT (1993). Detection of antisperm antibodies on the surface of living spermatozoa using flow cytometry: Preliminary study. J Fertil Steril.

[7] Haas GG, D'Cruz OJ, DeBault LE (1991). Comparisons of the indirect immunobead radiolabeled and immunofluorescence assays for immunoglobulin G serum antibodies to human sperm. J Fertil Steril.

[8] Denny TN, Scolpino A, Garcia A, Polyak A, Weiss SN, Skurnic JH (1995). Evaluation of T-lymphocyte subsets present in semen and peripheral blood of healthy donors: A report from heterosexual transmission study. J Cytometry.

[9] DeBaulny BO, LeVern Y, Kerboeuf D, Maisse G (1997). Flow cytometric evaluation of mitochondrial activity and membrane integrity in fresh and cryopreserved rainbow trout (Oncorhynchus mykiss) spermatozoa. J Cryobiol.

[10] DeBaulny BO, Labbe C, Maisse G (1999). Membrane integrity, mitochondrial activity, ATP content, and motility of the European catfish (Silurus glanis) testicular spermatozoa after freezing with different cryoprotectants. J Cryobiol.

[11] Adams SL, Hessian PA, Mladenov PV (2003). Flow cytometric evaluation of mitochondrial function and membrane integrity of marine invertebrate sperm. J Invertebr Reprod Dev.

[12] Molina J, Castilla JA, Gil T, Hortas MT, Vergara F, Herruzo A (1995). Influence of incubation on the chromatin condensation and nuclear stability of human spermatozoa by flow cytometry. J Mol Hum Reprod.

[13] Leonarda T, Antonio RMG, Andrea C, Galina K, Rita B, Gabriella P (1998). Mitochondrial membrane potential and DNA stainability in human sperm cells: A flow cytometry analysis with implications for male infertility. J Exp Cell Res.

[14] Zhu JC, Wang YX, Li JY (2007). Handbook of andrology diagnosis (in Chinese).

[15] World Health Organization (2001). Experimental inspection manual for human sperm-cervical mucus interaction (In Chinese).

[16] WHO (1992). WHO Laboratory Manual for Examination of Human Semen and semen-cervical mucus interaction.

[17] Evenson DP, Darzynkiewicz Z, Melamed MR (1982). Simultaneous measurement by flow cytometry of sperm cell viability and mitochondrial membrane potential related to cell motility. J Histochem Cytochem.

[18] Curi SM, Ariagno JI, Chenlo PH, Medeluk GR, Pugliensi MN, Sardi Segovia LM (2003). Asthenozoospenmaia; Analysis of a large population. J Arch Androl.

[19] O'Connell M, McClure N, Lewis SEM (2002). The effect of cryopreservation on sperm morphology, motility and mitochondrial function. J Hum Reprod.

[20] Galfano A, Novara G, Iafrate M, De Marco V, Cosentino M, D'Elia C (2009). Improvement of seminal parameters and pregnancy rates after antegrade sclerotherapy of internal spermatic veins. J Fertil Steril.

[21] Aziz DM, Enbergs H (2005). Stimulation of bovine sperm mitochondrial activity by homeopathic dilutions of monensin. J Homeopathy.

[22] Wu YM, Xia XY, Pan LJ (2006). Evaluation of sperm mitochondrial function using Rh123/PI dual fluorescent staining. Nat J Androl (in Chinese).

[23] Hauser R, Paz G, Botchan A, Yogev L, Yavetz H (2001). Varicocele and male infertility. Part II. Varicocele: effect on sperm functions. J Hum Reprod Update.

[24] Battaglia V, Salvi M, Toninello A (2005). Oxidative stress is responsible for mitochondrial permeability transition induction by salicylate in liver mitochondria. J Biol Chem.

[25] Jafari A, Zahmatkesh M, Sadeghipour HR, Kajbafzadeh A, Sarrafnejd A, Shahrestany T (2010). Flow cytometric evaluation of sperm superoxide anion production in rats with experimental varicocele. J Urol.

[26] Lu XY, Wu RSS (2005). Ultraviolet damages sperm mitochondrial function and membrane integrity in the sea urchin anthocidaris crassispina. J Ecotoxicol Environl Safety.

[27] Marchetti C, Obert G, Deffosez A, Formstecher P, Marchetti P (2002). Study of mitochondrial membrane potential, reactive oxygen species, DNA fragmentation and cell viability by flow cytometry in human sperm. J Human Reproduction.

[28] Henkel RR, Schill WB (2003). Sperm preparation for ART. J Reprod Biol Endocrinol.

